# Altruistic Attitudes Among Older Adults: Examining Construct Validity and Measurement Invariance of a New Scale

**DOI:** 10.1093/geroni/igaa060

**Published:** 2020-11-27

**Authors:** Tirth R Bhatta, Eva Kahana, Nirmala Lekhak, Boaz Kahana, Elizabeth Midlarsky

**Affiliations:** 1 Department of Sociology, University of Nevada, Las Vegas, US; 2 Department of Sociology, Case Western Reserve University, Cleveland, Ohio, US; 3 School of Nursing, University of Nevada, Las Vegas, US; 4 Department of Psychology, Cleveland State University, Ohio, US; 5 Department of Counseling and Clinical Psychology, Teachers College, Columbia University, New York, New York, US

**Keywords:** Altruistic love, Compassion, Kindness, Longitudinal invariance, Psychometrics

## Abstract

**Background and Objectives:**

Scholarly research has established the role of altruism in facilitating human cooperation and prosocial behaviors and highlighted its contribution to psychological well-being. Given the health significance of altruistic attitudes and orientations, we developed a valid and reliable measure of this construct that is suitable for use with older adults.

**Research Design and Methods:**

Based on data from a long-term panel study on adaptation to frailty among older adults (*n* = 366; mean age = 86 years), we used confirmatory factor analysis to perform construct validation of a five-item Elderly Care Research Center Altruism Scale among older adults (e.g., “Seeing others prosper makes me happy”). Moreover, we examined the invariance of the scale’s factor structure across time and gender using nested models.

**Results:**

Composite reliability (coefficient omega = 0.78), factor loadings (>0.45 with eigenvalue = 2.84) from exploratory factor analysis, and model fit indices (e.g., comparative fit index = 0.999) from confirmatory factor analysis suggest a single factor, supporting a unidimensional reliable construct of altruistic orientation at baseline. The results provided support for configural, metric, and scalar invariance across time. Findings pertaining to measurement invariance across gender confirmed full configural invariance but only offered support for partial metric, scalar, and residual invariance at baseline. Strong correlations among the altruism scale, salient personality traits, psychological well-being, religiosity, and meaning in life help establish construct validity.

**Discussion and Implications:**

The availability of a reliable and valid measure of altruistic attitudes enables a comprehensive evaluation of altruism’s influence on later-life health and well-being.

**Translational Significance:** We constructed a brief, reliable, and valid altruism scale that is useful for assessing this important prosocial orientation and resource among older adults and other age groups. The availability of a brief altruism scale with strong psychometric properties can be useful for planning programs and policies that utilize older adults’ potential for making contributions to society. Recognizing older adults’ generosity of spirit and desire to help others can also counteract ageist attitudes.

## Background

Every man must decide whether he will walk in the light of creative altruism or in the darkness of destructive selfishness [sic].—Martin Luther King, Jr.

Hypermaterialism and the consequent reduction of everyday relationships to mere economic considerations have given rise to concerns of eroding social solidarity (Derber, 2006; Ricard, 2015). The simultaneous increase of unfettered individualism and self-centeredness is likely to solidify “perceptions of self as atomistic individual as opposed to self as integrated with humanity as a whole” (Ambrose, 2009, p. 50). The empirical investigation of the impacts of the aforementioned societal changes on social solidarity and, subsequently, on psychological well-being requires a scale that can capture one’s concerns for fellow humans. Prior studies on the health benefits of altruism have observed its positive influence on later-life psychological well-being, with helping behaviors mediating some portion of such influence (Dulin et al., 2001; Kahana et al., 2013; Midlarsky & Kahana, 2007). Despite growing interest in the health significance of altruistic orientations, surprisingly little attention has been given to developing valid and reliable measures of this construct that are suitable for use among older adults and other age groups. Measurement of altruism represents an important area of inquiry in the social sciences, given its association with human cooperation, helping others, and engagement in prosocial interactions (Krebs & Van Hesteren, 1994; Piliavin & Charng, 1990).

Sociologist and philosopher Auguste Comte coined the term altruism in 1830 to capture our selfless care for others. Altruism, derived from the Latin word “alter” (i.e., “other”), has been conceptualized as a “motivational state with the ultimate goal of increasing another’s welfare” (Batson & Shaw, 1991, p. 109). It may also be viewed as compassion for other human beings that is motivated by generativity (Erikson, 1968) and by the need for meaningful human connectedness, even toward the end of life (Kahana et al., 2012). Altruism is recognized as an essential motive for prosocial human behavior, reflecting concern for others’ welfare without expectation of material benefit (Dovidio et al., 2006; Post et al., 2007). Altruistic attitudes influence helping behaviors that represent proactive adaptations and contribute to positive outcomes, even in the face of normative stressors (Kahana et al., 2012). Older adults participating in contributory activities can compensate for the loss of social ties due to bereavement (Hansson & Stroebe, 2007). Such ties are critical for life satisfaction and psychological well-being in later life (Morrow-Howell, 2010).

Among older adults, altruistic behaviors are exemplified in volunteering, making charitable donations, and offering informal assistance to those in need (Midlarsky & Kahana, 2007). It is important to note that even older adults who cannot engage in overt prosocial behaviors may exhibit altruistic beliefs and attitudes. Indeed, altruistic attitudes are likely motivating factors in late-life prosocial behaviors and make unique and positive contributions to the maintenance of positive affectivity in late life (Carlo et al., 2005; Kahana et al., 2013). As these findings indicate, the lack of engagement in prosocial behaviors for reasons beyond the individual’s control, such as poor health, signifies the unique salience of altruistic attitudes.

In light of the importance of altruism as a concept, it is surprising that in the prior literature, little attention has been directed to developing valid and reliable measures of attitudinal components of this construct, particularly among older adults. While laboratory and natural experiments have been adopted to measure altruism (Harbaugh & Krause, 2000), previous research has noted several drawbacks. A participant’s response to a particular stimulus (e.g., interaction with other participants in closely monitored games) in the laboratory environment likely differs from everyday interactions. The generalizability of inferences about altruistic behaviors drawn from small experimental samples that undergo a substantial degree of scrutiny has been pointed out as a limitation of experimental approaches (Levitt & List, 2007; Smith, 2006). Moreover, the practical difficulties of observing altruistic behaviors in a natural context are compounded by the even more challenging task of parsing out factors that lead to such behavior. Such problems have given credence to the usefulness of self-reports of altruistic attitudes and behaviors (Sawyer, 1966).

Self-report assessments hold great promise for advancing social science research on altruism among older adults. Previous studies assessed altruism through a mixture of self-reported attitudes and behaviors, but these studies focused on younger adults—typically university students—in South Korea (Lee et al., 2003), Germany (Büssing et al., 2013), and the United States (Nickell, 1998; Smith, 2006). Currently, no altruism scale has been validated among older adults. The best effort to measure altruism in adults was undertaken by Rushton et al. (1981) and resulted in the development of the Self-Report Altruism Scale. This 20-item scale combines the personal traits and altruistic acts of university students in Canada. It has good psychometric properties and has been used in subsequent studies (Eisenberg & Miller, 1987; Penner et al., 1995). However, this scale is limited to self-reporting of engagement in altruistic behaviors. For consideration of altruism in older adults, it is necessary to develop measures that can tap attitudes and self-concepts that are separate from behaviors. Moreover, existing studies fail to assess the equivalence of scale properties across time (i.e., longitudinal invariance) and gender (see Supplementary Table S1 for details). To appropriately examine substantial differences in altruism over time and across groups, it is important to establish that such group differences are not driven by differential responses to scale items. The lack of prior evidence on measurement invariance of altruism precludes understanding the ways different social groups may assign different meanings to the indicators of altruistic attitudes.

Altruism scales should have generalizability to other age groups as well and prove useful in assessing altruistic attitudes that are separate from behaviors, such as volunteering. Furthermore, brief scales hold additional advantages of being user-friendly and suitable for clinical and practice settings (Diener et al., 2010; Zimmerman et al., 2006). Given the significance of altruism for the health and well-being of older adults, a clear need exists for a validated scale representing different dimensions of altruistic attitudes (e.g., compassion for others, selflessness) to provide a comprehensive understanding of them. By incorporating a range of altruistic orientations, such a scale is likely to better capture altruism among older adults. Hence, it will provide a more accurate estimate of the influence of altruistic attitudes on health and well-being in later life. This study engages in such an endeavor by assessing the reliability and validity of a new altruism scale. The following research questions guide our study:

(1) Is the Elderly Care Research Center (ECRC) Altruism Scale internally consistent? Does one factor represent the items intended to measure altruistic attitudes?
(2) Does one factor provide the best fit for observed altruistic attitudes? Does the factorial structure of the altruism scale remain invariant across time? Are scale properties invariant across gender?
(3) Is altruism related to salient antecedents, such as personality traits and religiosity, and potential sequelae such as meaning in life and psychological well-being?

## Data and Methods

This study derives data from a panel study of successful aging, conducted by ECRC. Initiated in 1989, the panel study conducted annual in-person interviews to gather information on the later-life adaptation of retirement community–dwelling older adults (Kahana et al., 2002). The retirement community, located on the West Coast of Florida, attracts active and healthy older adults. We focus on Waves 10 and 13 of the data due to the availability of items administered to measure altruism. At the onset of the study, we selected 1,000 older adults who were free from significant physical and psychological health problems and living in Florida at least 9 months of the year to participate in this study. These individuals were selected from 3,905 households randomly drawn from the residential listing provided by the retirement community. Excluding 48.9% of households (i.e., 1,909) that did not meet eligibility criteria, we conducted in-person face-to-face interviews with older adults from 908 households. The structured interviews conducted annually for 22 years lasted for 60–70 min. The response rate at baseline for those invited to participate in this study was 77.3%.

We based our analysis on 366 respondents who reported their altruistic orientation at Wave 10. Attrition of respondents resulted primarily from death with a loss to follow-up (e.g., respondent moved or lost interest) contributing only 7% to annual attrition. The respondents who died or left the study were more likely to be male, unmarried, and older than those who remained in our study. Given our relatively small sample, we used a single imputation procedure to estimate missing observations on altruism indicators at Wave 13.

### Measures

#### Altruism

This study uses five self-reported altruistic attitude items to construct the new altruism scale. These items, administered at Waves 10 and 13, were developed to capture selflessness, generosity of spirit, and willingness to help. We adapted our scale items from Wrightsman’s (1964) Philosophy of Human Nature Altruism Scale that was designed to measure “unselfishness, sincere sympathy, and concern for others” (p. 744). We framed items in our altruism scale as reports about the self rather than generalizations about other people. Items referring to attitudes rather than behaviors were selected and presented in a simplified and unambiguous fashion. For instance, the altruism scale item “I really care about the needs of other people” was adapted from the Wrightsman scale’s “The typical person is sincerely concerned about the problems of others.” We asked respondents on a 5-point Likert scale (i.e., *strongly disagree* = 1, *disagree* = 2, *neither agree nor disagree* = 3, *agree* = 4, *strongly agree* = 5) to what extent they agreed with five statements. These statements include (Altruism 1) “I enjoy doing things for others,” (Altruism 2) “I try to help others, even if they do not help me,” (Altruism 3) “Seeing others prosper makes me happy,” (Altruism 4) “I really care about the needs of other people,” and (Altruism 5) “I come first and should not have to care so much for others.” To aid interpretation, we reverse-coded responses to the Altruism 5 item to compare them with other statements. We obtained factor scores to represent a respondent’s altruism at Wave 10 by performing a confirmatory factor analysis (CFA) with a robust maximum likelihood (ML) estimation procedure. These factor scores were used to estimate correlation coefficients to assess the construct validity of the altruism scale.

#### Mental health sequelae of altruism

Prior research has documented the positive influence of altruistic attitudes on older adults’ psychological well-being, reflected in reduced depressive symptoms, more positive and fewer negative affects (Kahana et al., 2013). This finding is consistent with prior research documenting the mental health benefits of altruism (Midlarsky & Kahana, 2007). Specific findings relate to greater life satisfaction among those with altruistic orientations (Dulin et al., 2001). Such findings support more generalized expectations about health benefits and enhanced goals and meaning in life (Post, 2007). Meaning in life has been increasingly studied as a cornerstone of psychological well-being (Heintzelman & King, 2014). Research focusing on life course levels and correlates of meaning in life has identified higher levels of meaning among older respondents (Steger et al., 2009).

#### Positive and negative affect

We used the Positive and Negative Affects Schedule scale to measure positive and negative affect (Watson et al., 1988). Among 10 words used to capture different emotions, five words describe positive affects (e.g., happy, alert) and five items describe negative affects (e.g., afraid, nervous). On a 5-point scale, respondents were asked to report the extent to which they felt specific emotions during the past year. We constructed two scales (positive and negative affects) by summing responses across items. A higher score for each affect scale reflects greater affect levels.

#### Depressive symptomatology

We used the 10-item short version of the Center for Epidemiological Studies—Depression scale to assess depressive symptoms (Andresen et al.,1994). A series of questions were employed to inquire about the frequency of feeling specific emotions (e.g., “had the blues”). The responses were recorded on a 5-point scale, with responses ranging from 1 (*never or rarely*) to 5 (*all the time*). We summed the items to create a single score, with higher scores representing greater depressive symptomatology.

#### Goals and meaning in life

We measured respondent goals in life by asking them to report to what extent they have goals or aims. The responses were recorded on a 5-point scale, ranging from 1 (*no goals or aims*) to 5 (*very clear goals or aims*). Their assessment of meaning in life was captured by inquiring how meaningful they found their existence to be. Responses recorded at Wave 10 ranged from 1 (*utterly meaningless*) to 5 (*very meaningful*).

### Antecedents of Altruistic Attitudes

#### Religiosity

Most religious orientations extol the value of altruism or selfless giving (Zhao, 2012). Studies based mostly on young respondents have confirmed an association between religious beliefs and practices and altruistic attitudes (Pessi, 2011). We asked respondents a question intended to evaluate how religious they considered themselves. Their responses, recorded at Wave 10, ranged from 1 (*not at all religious*) to 5 (*very religious*).

#### Personality

Personality has been frequently noted as being associated with altruism. Some scholars have gone as far as describing altruistic orientations as constituting an altruistic personality (Rushton et al., 1981). Altruistic orientations have been linked to extraversion, as measured by the Eysenck Personality Questionnaire (Rushton et al., 1989). In more recent studies, personality was also found to be associated with altruism in daily life (Oda et al., 2014). We used four personality traits (kind, sympathetic, envious, and cold) from the Big-Five Inventory to examine the relationship between each personality trait and altruism. Respondents at Wave 8 were asked to report how well each personality trait applied to them. Their response ranged from 1 (*not at all*) to 5 (*very much*).

### Analytic Plan

Our analyses proceeded in three stages by using both SAS 9.4 and Mplus version 8. The first stage involved estimating correlation coefficients to examine interrelationship among variables that measure altruistic attitudes. We considered the correlation coefficient of at least moderate strength (i.e., *r* ≥ 0.30) to indicate substantial interrelationship among scale items. We calculated McDonald’s coefficient omega to further assess internal consistency (i.e., reliability) of scale items measuring altruism (McDonald, 1999). The calculation of coefficient omega was based on factor loadings and error variances that were derived from the CFA of our five-item altruism scale. We considered items with a value of at least 0.70 to be strongly interrelated. We identified factorial structure among scale items by performing exploratory factor analysis (EFA) with oblique rotation (e.g., GEOMIN). We used factor loadings greater than 0.40 as the threshold to select items that reflect altruism.

In the second stage, we performed CFA to assess the fit of the altruism scale’s factorial structure with our data by using robust ML estimation. The mean- and variance-adjusted maximum likelihood estimation approach employed in this study adjusts for multivariate nonnormality to generate robust standard errors and a mean- and variance-adjusted chi-square test of model fit. This estimation approach has performed better than the mean- and variance-adjusted weighted least squares estimation when the sample size is small (Li, 2016). We considered our CFA model fit to be acceptable if fit indices satisfied commonly accepted thresholds for the comparative fit index (CFI), the Tucker–Lewis index (TLI; i.e., close to 0.95 or greater), and the root mean square error of approximation (RMSEA; i.e., close to 0.06 or below; Brown, 2015; Hu & Bentler, 1999).

After examining the fit of the altruism scale’s factorial structure, we assessed the equivalence of scale properties separately across time and gender by performing measurement invariance tests in sequential (or nested) steps. Our single-level, cross-time model entailed placing invariance constraints across time, whereas a multiple-group approach required us to place such constraints across gender. We accounted for the dependence of repeated observations over time by allowing residual covariances between the same indicators across time. Our nested models examined four types of measurement invariance: configural, weak factorial (i.e., metric), strong factorial (i.e., scalar), and strict (i.e., residual invariance; Byrne et al., 1989; Widaman & Reise, 1997). Embodied in our baseline model, configural invariance requires the same factorial structure to exist across time and gender. We allowed all factor loadings, item intercepts, and residuals to vary across time and gender in this step. Our second model, nested within the baseline model, examined weak factorial invariance by constraining factor loadings to be equivalent while allowing all items intercepts and residuals to be freely estimated across time and gender. We continued investigating measurement invariance by testing the equivalence of item intercepts across time and gender. This test to assess strong factorial invariance builds on the metric invariance model by constraining both factor loadings and item intercepts to be equal but allowing item residuals to vary across time and gender (Vandenberg & Lance, 2000). In the last step, we examine strict measurement invariance by constraining all factors loadings, item intercepts, and residuals to be equal across time and gender. Each of the aforementioned measurement invariance models freely estimated factor covariances and residual covariances for all items across time and gender.

We examined measurement invariance by comparing fit indices between two nested models. The latter model contained more equality constraints on parameters across time and gender than the previous model. Using difference testing (DIFFTEST) with the Satorra–Bentler scaled chi-square statistic (SB-χ ^2^), we compared the difference in fit between two nested models (e.g., lower constraints vs. a more restrictive model). We considered it a confirmation of measurement invariance if constraining additional parameters across time and gender led to a poorer fit. As a result of constraining parameters, the better fit was considered as evidence of noninvariance. Such evidence was used to stop our analysis from proceeding to the next step. The decrease in the value of comparative fit indices (e.g., a decrease in CFI of ≥0.01) and an increase in the value of indices that represent model parsimony (e.g., an increase in RMSEA of ≥0.015) reflected a poorer fit and, hence, was used as evidence for measurement invariance (Chen, 2007). Finally, we assessed construct validity by examining the correlation between altruism factor scores and theoretically similar constructs (e.g., agreeableness, a personality disposition). We gauged predictive validity by examining the correlation of altruism at Wave 10 with constructs such as positive and negative affects and depressive symptoms at Wave 13.

## Results

We present the demographic characteristics of respondents in Table 1. The average age of our respondents was 86 years, with ages ranging from 70 to 105 years. Sixty-eight percent of respondents were women, and 38% were married. Our respondents were relatively well educated, with about 25% of them having education beyond high school. Our evaluation of interrelationships among indicators of altruistic orientation began with assessing correlation coefficients at Wave 10. Table 2 presents that correlation coefficients ranged from 0.31 to 0.71, which suggested substantial interrelationship among scale items. The strong interrelationship is further illustrated by high coefficient omega at Wave 10 (0.78) and Wave 13 (0.81).

**Table 1.** Demographic Characteristics of Study Respondents, Elderly Care Research Center Florida Retirement Study (1999–2002)

Variables of interest	Mean (*SD*) or percentage (*n* = 366)
Age (range: 70–105 years)	86.17 (4.53)
Gender (1 = *Woman*)	68.31
Marital status (1 = *Married*)	38.36
*Education*	
Less than high school	38.71
High school	36.45
Beyond high school	24.84
Religiosity (range: 1–5)	3.56 (0.95)
*Personality traits*	
Kind (range: 1–5)	4.16 (0.80)
Sympathetic (range: 1–5)	4.26 (0.81)
Envious (range: 1–5)	1.24 (0.57)
Cold (range: 1–5)	1.37 (0.71)
Goals in life (range: 1–5)	2.34 (1.52)
Meaning in life (range: 1–5)	3.81 (1.07)
Positive affect (range: 5–25)	15.42 (3.61)
Negative affect (range: 5–25)	9.91 (3.39)
Depressive symptoms (range: 10–50)	21.60 (5.58)

**Table 2.** Correlation Among Items Measuring Altruism at Wave 10

Scale items	Altruism 1	Altruism 2	Altruism 3	Altruism 4	Altruism 5
Altruism 1	1				
Altruism 2	0.45***	1			
Altruism 3	0.55***	0.49***	1		
Altruism 4	0.44***	0.45***	0.71***	1	
Altruism 5	0.40***	0.34***	0.38***	0.31***	1

*p < .05, **p < .01, ***p < .001.

Results from EFA presented in Table 3 helped identify factorial structure among scale items. We obtained strong initial evidence for the existence of a single factor at both waves because only one factor had an eigenvalue greater than 1 (2.84 at Wave 10; 2.97 at Wave 13). All items were strongly loaded in one factor at Wave 10, with factor loadings ranging from 0.46 to 0.88. Except for a slight difference in factor loadings for two items between the two waves (Altruism 2: 0.59 at Wave 10 vs. 0.81 at Wave 13; Altruism 3: 0.88 at Wave 10 vs. 0.74 at Wave 13), all other items demonstrated similar factor loadings at Wave 13. Following EFA, we performed CFA to examine whether our hypothesized one-factor model fit the baseline data. Our fit indices (not presented in Table) for the CFA model (χ ^2^(4) = 4.36, *p* = .36, CFI = 0.999, TLI = 0.998, RMSEA = 0.016, *p*(RMSEA) = .723) demonstrate excellent fit to the data at Wave 10.

**Table 3.** Exploratory Factor Analysis

	Factor loadings	
Scale items	Wave 10	Wave 13
Altruism 1	0.63	0.72
Altruism 2	0.59	0.81
Altruism 3	0.88	0.74
Altruism 4	0.79	0.74
Altruism 5	0.46	0.48

### Longitudinal Invariance

After establishing the one-factor model’s fit to the data at baseline, we proceeded to test whether the same factorial structure is maintained across waves. Table 4 displays model fit indices from a series of nested models designed to examine longitudinal invariance. As evidenced by model fit indices (χ ^2^(27) = 28.422, *p* = .389; CFI = 0.997, TLI = 0.995, RMSEA = 0.012, *p*(RMSEA) = .984), the configural invariance model (i.e., unconstrained model) also provided excellent fit to our data. Our comparison between the metric invariance and configural invariance models via the DIFFTEST procedure indicates a statistically not significant difference between those two models (ΔSB-χ ^2^(4) = 4.929, *p* = .295). The statistically not significant difference in the SB-χ ^2^ statistic and negligible decrease in CFI between configural invariance and metric invariance models suggest that constraints in parameters imposed by the latter model did not lead to a significant worsening of model fit. Hence, the equality constraints imposed on the factor loadings across time were validated.

**Table 4.** Model Fit Indices From Tests of Longitudinal Invariance

Measurement invariance	*df*	SB-χ ^2^	ΔSB-χ ^2^	*p*_ΔSB_	CFI	TLI	RMSEA	*p*_(RMSEA)_
Configural invariance	27	28.422, *p* = .389	—	—	0.997	0.995	0.012	.984
Weak factorial (metric) invariance	31	32.791, *p* = .379	4.929	.295	0.996	0.994	0.013	.989
Strong factorial (scalar) invariance	36	38.275, *p* = .367	12.426	.029	0.995	0.994	0.013	.993

*Note: df* = degrees of freedom; SB-χ ^2^ = Satorra–Bentler chi-square statistic; CFI = comparative fit index; TLI = Tucker–Lewis Index; RMSEA = root mean square error of approximation.

Given the evidence in support of metric invariance, we placed additional constraints on parameters across time. We constrained item intercepts to be equal across time to examine scalar invariance. This model’s fit indices were significantly worse than the metric invariance model, as shown by significantly different SB-χ ^2^ statistics between two nested models (ΔSB-χ ^2^(5) = 12.426, *p* = .029). Additional analysis showed that the model fit improved (ΔSB-χ ^2^(3) = 8.293, *p* = .04) after allowing intercepts for two items (Altruism 3 and Altruism 4) to freely estimate across time. We decided not to pursue further analysis to explore partial scalar or strict (i.e., residual) invariance for the following two reasons. First, the changes in other model fit indices were within acceptable margins for the confirmation of scalar invariance (i.e., 0.001 decrease in CFI and no change in RMSEA between metric and scalar invariance models). Evidence of strong measurement invariance is sufficient to engage in meaningful comparisons of latent means of altruism across time. Second, we do not have theoretical justifications or previous guidance on which item intercepts to place further constraints.

### Measurement Invariance Across Gender

In addition to longitudinal invariance, we assessed invariance of scale properties across gender at baseline. Supplementary Table S2 presents model fit indices from a series of nested models designed to examine measurement invariance across gender. Our model fit indices for the configural invariance model (i.e., unconstrained model; χ ^2^(8) = 4.83, *p* = .775; CFI = 1.00, TLI = 1.023, RMSEA = 0.000, *p*(RMSEA) = .921) indicate excellent fit to our data. Our metric invariance model, which placed model constraints on factor loadings across gender, led to a decrease in model fit. The significant decrease in fit relative to the configural invariance model (ΔSB-χ ^2^(4) = 15.72, *p* = .007) suggests a lack of metric invariance. We examined partial metric invariance by allowing factor loadings of the Altruism 1 variable to differ across gender. Our decision was guided by EFA findings that showed substantially greater factor loading for men (0.84) relative to women (0.54, not presented in Table). Greater factor loading for men indicates that they are more likely to identify with this item than women. Our partial metric invariance model did not result in a significantly different fit (ΔSB-χ ^2^(3) = 7.59, *p* = .087) than the configural invariance model. In other words, the model fit after relaxing constraints on factor loadings for the Altruism 1 variable did not significantly worsen, which offers support for the partial metric invariance.

We fit additional models to examine scalar and residual invariance across gender. For both models, we kept factor loadings for the Altruism 1 variable unconstrained across gender in our tests for the next levels of invariance. We examined scalar invariance by constraining item intercepts to be equal across gender. This model’s fit indices were not significantly worse than the partial metric invariance model (ΔSB-χ ^2^(4) = 4.14, *p* = .346). Finally, we placed additional constraints on residuals across gender. We did not observe a significant worsening of model fit for this model (ΔSB-χ ^2^(5) = 3.65, *p* = .445) relative to the partial scalar invariance model. The lack of significant worsening of model fit suggests support for both partial strong factorial and strict factorial invariance.

### Assessment of Construct Validity

We present results from our analysis of construct validity in Table 5. The altruism scale was negatively correlated with self-evaluations such as “envious” (*r* = −0.19, *p* < .01) and “cold” (*r* = −0.35, *p* < .001, but was positively correlated with the self-concepts of sympathy (*r* = 0.26, *p* < .001) and kindness (*r* = 0.30, *p* < .001). Respondents with higher altruism scores were significantly less likely to report themselves as envious and cold; they were more likely to consider themselves as kind and sympathetic. We observed statistically significant correlations between the altruism scale and psychological well-being. Findings suggested significantly lower depressive symptoms (*r* = −0.29, *p* < .001) and higher positive affect (*r* = 0.37, *p* < .001) among respondents with higher altruism scores. Although negative correlation indicates a lower negative affect among those with higher altruism, the correlation coefficient was not statistically significant (*r* = −0.07, *p* = .19). Strong positive correlations between altruism and religiosity (*r* = 0.30, *p* < .001), goals (*r* = 0.31, *p* < .001), and meaning in life (*r* = 0.30, *p* < .001) were also documented.

**Table 5.** Construct Validity

	Correlation coefficient
Variables	Altruism Wave 10
Religiosity	0.30***
Kind	0.30***
Sympathetic	0.26***
Envious	−0.19**
Cold	−0.35***
Goals in life	0.31***
Meaning in life	0.30***
Positive affect	0.37***
Negative affect	−0.07
Depressive symptoms	−0.29***

*p < .05, **p < .01, ***p < .001.

## Discussion

Our study addresses the lack of valid and reliable measures of altruistic attitudes, particularly among older adults, in the literature. The absence of such scales is particularly surprising, given the growing interest in the health significance of altruistic orientations. Furthermore, a brief altruism scale is easier to administer to older populations (Diener et al., 2010). Our study evaluated the reliability and validity of the ECRC’s five-item Altruism Scale. We also examined the invariance of the factorial structure of the altruism scale across time and gender. Our findings confirm the scale’s reliability, as indicated by a strong interrelationship among indicators of altruistic orientation. Significantly high factor loadings on a single factor supported a one-dimensional construct of altruism at both waves. Findings from a CFA model at baseline demonstrated excellent fit to the data, which further affirms our hypothesized one-factor model of altruism.

Our longitudinal invariance tests revealed support for configural, weak factorial (i.e., metric), and strong factorial (i.e., scalar) invariances across time. Our multiple-group invariance tests also confirmed the configural invariance across gender. However, findings from those tests only offered support for partial metric, scalar, and residual invariance at baseline. Confirmation of configural measurement invariance suggests that the overall representation of altruism by a single factor is consistent across time and gender. In other words, the invariance of factor loadings shows that the ECRC’s five-item Altruism Scale represents a single factor of altruism across time and gender. Support for the metric longitudinal invariance reflects that each item representing the altruism scale loads similarly into a single factor and with similar magnitude across time. The metric invariance is essential for comparing factor variances and covariances when studying altruism over time (Brown, 2015; Vandenberg & Lance, 2000).

We did not find support for the scalar longitudinal invariance based on the SB-χ ^2^ statistic. Relative to the metric invariance model, the model fit for the scalar invariance model worsened, indicating that some item intercepts could differ across time. In the context of our study, the violation of scalar invariance does not necessarily indicate systemic response bias across time. The differences in item intercepts may instead suggest valid changes in levels of altruism over time. It is plausible that mean levels of altruism may increase over time (Midlarsky & Hannah, 1989; Post, 2007). However, we affirmed scalar longitudinal invariance based on other model fit indices such as CFI and RMSEA. The changes in those indices were within the acceptable margins for the confirmation of measurement invariance (Chen, 2007). The decrease in CFI was lower than 0.01, whereas the values of RMSEA for both metric and scalar invariance models were identical. We considered this evidence to confirm scalar longitudinal invariance; thus, we did not examine strict (or residual) invariance across time. The scalar invariance of the altruism scale removes the possibility that differences in factor means of altruism over time could be due to differences in scale properties across waves. Even though strict factorial invariance (i.e., equivalence of item residuals along with factor loadings and item intercepts across time) has been considered to reflect a true measurement invariance (Blankson & McArdle, 2015; Vandenberg & Lance, 2000), scholars generally concur that confirmation of configural, metric, and scalar invariance meets sufficient criteria for establishing measurement invariance across time (Putnick & Bornstein, 2016; Widaman & Reise, 1997).

While we were unable to establish full metric invariance, additional measurement invariance models fitted identified the item “I enjoy doing things for others” as contributing to a lack of metric invariance across gender. The phrase “I enjoy doing things for others” implies instrumental action associated with masculinity (Bennett, 2007). Men would more readily identify with such actions than women. Consequently, it is more likely to indicate higher altruism in men than women. Conversely, a scale item could have indicated higher altruism in women than men, had the phrase been “I enjoy taking care of others.” The latter phrase appeals to the nurturing qualities of women. As expected, the relaxation of constraints on factor loadings for our non-invariant item helped us establish partial metric, scalar, and residual invariance across gender. Our findings encourage future research studies on gender differences in altruistic orientations to be mindful of gender-specific measurement differences concerning the phrase “I enjoy doing things for others.”

A strong correlation between the ECRC Altruism Scale, salient antecedents such as personality traits and religiosity, and potential sequelae such as psychological well-being and meaning in life establish construct validity for the altruism scale. Self-evaluations like “envious” and “cold” were significantly lower among older adults with higher altruism. By contrast, higher altruism was more likely to correspond with reports that expressed self-concepts like sympathy and kindness. These findings are consistent with prior literature regarding personality features (Oda et al., 2014) and religious orientations (Pessi, 2011) as important antecedents of altruism. Considering the sequelae of altruism, our findings are consistent with expectations based on prior research (Kahana et al., 2013). Except for negative affect, altruism was significantly associated with psychological well-being. Older adults with higher altruism reported lower depressive symptoms and higher positive affect. Findings also confirm expectations regarding significantly greater goals and meaning in life among older adults with higher altruism (Post, 2007).

Our scale’s good construct validity supports its utility for relating altruistic attitudes to theoretically salient antecedents and sequelae. The development of short, reliable, and valid indicators of altruistic attitudes is consistent with Diener et al.’s (2010) efforts to construct short and easy-to-administer well-being measures. We argue that a reliable and valid measure of altruism will enable a comprehensive evaluation of altruism’s influence on health and psychological well-being in late life. We contend that our short altruism scale will be particularly useful for future studies on older adults. A short scale will reduce the time needed to complete a survey. Given that older adults are more likely to face health problems, less time to complete a survey is preferable (Diener et al., 2010). Limitations of our scale development efforts in this study include having a racially homogeneous sample who are reasonably well educated. Thus, we do not know if the ease of administration may differ for older adults with limited education. We do not consider this a major problem because the scale has good face validity and does not call for understanding complex concepts. Thus, we are proposing this newly developed and validated brief scale, the ECRC Altruism Scale, as a promising and useful measurement instrument in the field of altruism research with older populations. As there are growing numbers of older adults attaining old-old age, it is important to expand our measurement strategies to assess the positive potential of older adults for altruism and other qualities that counteract ageist stereotypes of late life. We also note that our scale is likely to have applicability to a broad range of age groups, despite being validated in a sample of older adults. Future research should expand investigations of measurement invariance of altruistic orientations across age (e.g., younger age groups), socioeconomic, and racial/ethnic groups to generate confirmatory evidence in that regard.

## Supplementary Material

igaa060_suppl_Supplementary_Materials
