# Supplementary material for: Altruistic Attitudes Among Older Adults: Examining Construct Validity and Measurement Invariance of a New Scale
Source: Innov Aging. 2020 Nov 27;5(1):igaa060. doi: 10.1093/geroni/igaa060 (PMC8631078; doi:10.1093/geroni/igaa060)
Supplement: igaa060_suppl_Supplementary_Materials [file igaa060_suppl_Supplementary_Materials.docx]

**Table S1.** Summary of Existing Altruism Scales

| Scale | Consruct | Study population | Ages | Measurement invariance Test | Number of items | Representative  items |
| --- | --- | --- | --- | --- | --- | --- |
| Philosophies of Human Nature Altruism Scale (Wrightsman Jr, 1964) | Beliefs about altruism | *n=*530 university students, US. | Not reported | No | 14 | “The typical person is sincerely concerned about the problems of others.” |
| Self-Report Altruism Scale (SRA; Rushton et al., 1981) | Altruistic behaviors | *n*=118 undergraduate students, Canada | Not reported | No | 20 | “I have given money to a charity.” |
| Helping Attitude Scale (HAS; Nickell, 1998) | Helping attitudes and behaviors | *n*=408 undergraduate students, US. | Not reported | No | 20 | “Helping friends and family is one of the great joys in life.  I donate time or money to charities every month.” |
| Altruism Scale for Adults (Lee et al., 2003) | Altruistic predisposition | *n*=592 (340 university students and 252 people from the general population, South Korea) | 20-35 years | No | 28 | "Do unto othersas you would have them do unto you is a motto I follow" |
| Generative Altruism Scale (GALS; Büssing et al., 2013) | Altrustic attitudes and behaviors | *n*=873 adolescents/  young adults, Germany | Mean= 19.1 years | No | 7 | “When I see suffering, I try tofind ways toalleviate it.  When I see individuals in need, I ask them how I can help.” |

**Table S2.** Model Fit Indices From Tests of Measurement Invariance

| Measurement Invariance | df | SB-χ^2^ | ΔSB-χ^2^ | p_ΔSB_ | CFI | TLI | RMSEA | p_(RMSEA)_ |
| --- | --- | --- | --- | --- | --- | --- | --- | --- |
| Configural Invariance | 8 | 4.83  p=0.775 | -- | -- | 1.00 | 1.023 | 0.000 | 0.921 |
| Weak Factorial  (i.e., metric) Invariance | 12 | 20.55  p=0.057 | 15.72 | 0.007 | 0.975 | 0.958 | 0.063 | 0.287 |
| Partial Weak Factorial (i.e., metric) Invariance | 11 | 12.42  p=0.332 | 7.59 | 0.087 | 0.996 | 0.992 | 0.027 | 0.676 |
| Partial Strong Factorial (i.e., scalar) Invariance | 15 | 16.56  p=0.346 | 4.14 | 0.391 | 0.995 | 0.994 | 0.024 | 0.737 |
| Partial Strict Factorial (i.e., residual) Invariance | 20 | 20.207  p=0.445 | 3.65 | 0.504 | 0.999 | 0.999 | 0.008 | 0.848 |

*Notes.* df--degrees of freedom; SB-χ2--Satorra-Bentler chi-square statistic; CFI--comparative fit index; TLI--Tucker Lewis Index; RMSEA--root mean square error of approximation
